# Deep Autoencoder Neural Networks for Short-Term Traffic Congestion Prediction of Transportation Networks

**DOI:** 10.3390/s19102229

**Published:** 2019-05-14

**Authors:** Sen Zhang, Yong Yao, Jie Hu, Yong Zhao, Shaobo Li, Jianjun Hu

**Affiliations:** 1 Chengdu Institute of Computer Application, Chinese Academy of Sciences, Chengdu 610041, China; sen.zhang@gmail.com; 2 University of Chinese Academy of Sciences, Beijing 100049, China; 3 School of Big Data, Guizhou Institute of Technology, Guiyang 550003, China; 4 School of Mechanical Engineering, Guizhou University, Guiyang 550003, China; yao_yong92@163.com; 5 College of Big Data Statistics, GuiZhou University of Finance and Economics, Guiyang 550025, China; jason.houu@gmail.com; 6 Department of Computer Science and Engineering, University of South Carolina, Columbia, SC 29201, USA; yongz@email.sc.edu

**Keywords:** transportation network, traffic congestion forecasting, spatial-temporal correlation, deep learning, end-to-end, deep autoencoder, convolutional neural network, long short-term memory

## Abstract

Traffic congestion prediction is critical for implementing intelligent transportation systems for improving the efficiency and capacity of transportation networks. However, despite its importance, traffic congestion prediction is severely less investigated compared to traffic flow prediction, which is partially due to the severe lack of large-scale high-quality traffic congestion data and advanced algorithms. This paper proposes an accessible and general workflow to acquire large-scale traffic congestion data and to create traffic congestion datasets based on image analysis. With this workflow we create a dataset named Seattle Area Traffic Congestion Status (SATCS) based on traffic congestion map snapshots from a publicly available online traffic service provider Washington State Department of Transportation. We then propose a deep autoencoder-based neural network model with symmetrical layers for the encoder and the decoder to learn temporal correlations of a transportation network and predicting traffic congestion. Our experimental results on the SATCS dataset show that the proposed DCPN model can efficiently and effectively learn temporal relationships of congestion levels of the transportation network for traffic congestion forecasting. Our method outperforms two other state-of-the-art neural network models in prediction performance, generalization capability, and computation efficiency.

## 1. Introduction

Onsets of freeway congestion reduce efficiency and capacity of transportation networks and should be forecast to take measures to prevent its formation in an accurate and timely manner in most situations [1,2]. Compared with flow or volume prediction [3,4,5,6], traffic congestion forecasting is more intuitive to both road travelers and transportation administrators. It can help road travelers to make better route selections, reduce pollution such as emission of greenhouse gases, and improve transportation operation efficiency. For these reasons, many prediction methods have been proposed and evaluated for traffic congestion prediction [7,8,9].

Various kinds and amounts of traffic data have been used by researchers in recent years for traffic condition prediction and related research of intelligent transportation systems. Most of these works use data sources such as road sensors, induction loops, automatic vehicle identification systems, remote traffic microwave sensors, in-road reflectors, floating car data, and simulation [3,4,10,11,12,13]. Images from cameras installed on roads, aerial photographs and remote sensing images as another kind of data source have also been used [14,15]. These kinds of data are either difficult to access due to special requirement of permissions or often outdated. On the other hand, many traffic administrative departments [16,17] and online map service providers [18,19,20] provide real-time or near real-time online traffic congestion condition maps to the general public for free. Such services use and integrate various data sources, for example induction loops and more generally location-based data originating from apps active on Global Positioning System (GPS)-enabled smartphones carried in running cars. Such maps provide a new kind of real-time traffic condition data source which differs vastly in timeliness, accessibility, availability, and coverage from aforementioned kinds of data sources. Yet there are few studies based on such data sources for traffic congestion prediction. This paper proposes a systematic method to collect and use this new kind of data source for traffic congestion forecasting.

Furthermore, traffic congestion maps provided by traffic administration departments and online map services often cover areas with road networks of various scales instead of a single road. It is more significant and interesting to understand and forecast traffic congestion in an entire road network rather than on a single road. It is also more helpful to present complete traffic congestion information for travelers to choose routes wisely and for traffic administrators to manage road networks and allocate resources systematically. However, it is more demanding to predict congestion for a road network and thus needs abilities to deal with more computational complexity introduced by the network topology, to generate more timely and effective prediction from a three-dimensional perspective, and to output more accurately predicted congestion levels. Unfortunately, conventional traffic congestion prediction models, which represent each road or road segment as an element in a sequence ignoring impacts of road length [7] or only consider a limited number of road links, do not meet aforementioned requirements due to limitations in input and output representation and normalization, improper hypotheses and assumptions, and inability to cope with the curse of dimensionality [21]. Thus, existing models may fail to predict large-scale network traffic congestion.

Motivated by hierarchical feature extraction and motion prediction, this paper represents congestion levels of a traffic network as a 2D-matrix and introduces an image-based deep learning approach for congestion forecasting. A deep learning architecture inspired by deep autoencoders (DAE) [22] is proposed to first learn low-dimensional vector representations of features and relationships embodied in images obtained from aforementioned data sources and then predict future congestion levels in a supervised way based on these learned representations. An autoencoder is a neural network consisting of an encoding module (encoder) and a decoding module (decoder) designed to learn representational properties of the input data [23]. Furthermore, multiple encoders and decoders can transform high-dimensional data into low-dimensional vector representations for further applications [22,24,25].

This paper tries to forecast future congestion levels of a transportation network using only historical temporal information and correlations of congestion levels of the network. In particular, we examine whether it is feasible to directly predict and output road network congestion images through the examination of just a sequence of previous congestion images for that same transportation network in an end-to-end approach with the help of a simple yet efficient deep learning neural network architecture inspired by DAEs.

The contributions of the paper are summarized as follows:

- We propose an accessible and general approach to collect, transform, and represent snapshots of transportation network maps marked with traffic congestion conditions for roads inside, which are publicly available from transportation administrative departments and online traffic map service providers. Based on this approach, we have built and released a long-span traffic congestion dataset.
- We develop a deep neural network model for efficient end-to-end prediction of transportation network congestion levels by using hierarchical feature extraction. Our end-to-end learning model directly outputs prediction results presented visually and intuitively in the same road network structure and form as inputs are, thus eliminating the need for manual feature selection and engineering.
- Our extensive experiments on a transportation network in the Seattle area demonstrates effectiveness and efficiency of the proposed approach.

The remainder of this paper is organized as follows: Section 2 discusses the related literature on traffic prediction. Section 3 presents a systematic approach to collect and transform snapshots of transportation network maps with congestion levels, introduces a grid-based representation method for traffic congestion levels, and shows the architecture of our deep neural network to learn temporal feature representation and correlations for traffic congestion prediction. In Section 4 a transportation network in the Seattle area, Washington state is used to build a traffic congestion dataset and to test the effectiveness of the proposed model. To evaluate the performance of our proposed neural network model, we compare two state-of-the-art deep learning models titled convolutional long short-term memory (LSTM) Network (ConvLSTM) [26] and Spatiotemporal Recurrent Convolutional Network (SRCN) [6]. Then in Section 5 we discuss implications and limitations of our work. The conclusions and future studies are presented in the final section.

## 2. Related Work

There are two main categories of approaches for prediction of traffic related variables such as speed, volume, and density, namely parametric and nonparametric approaches [21,27].

Being a parametric approach, the Auto Regressive Integrated Moving Average (ARIMA) model was proposed to construct models from time series of historical states to predict future values. Parameters of the ARIMA model could be configured via the Box-Jenkins method [28]. Smith and Williams used the ARIMA model for the first time to predict traffic flow at a single point [28]. A family of ARIMA-based models, such as seasonal ARIMA (SARIMA) models [29,30,31], KARIMA models [32], ARIMAX models [33], and CTM-SARIMA models [34], have been deployed for traffic forecasting since then. These parametric approaches share common requirements which demand predetermined structures of models according to theoretical or physical assumptions and tuning a set of parameters to reflect the evolution of traffic conditions in the real world as much as possible [35,36]. Especially SARIMA models are found to incur high computational cost [28].

The limitations of parametric algorithms have led the shift to nonparametric approaches for traffic prediction such as nonparametric regression, Support Vector Machines (SVM), and Artificial Neural Networks. As a nonparametric approach, k-nearest neighbors (KNN) models have been used to forecast traffic speeds and flows, with both univariate and multivariate cases [37,38,39,40,41]. Supporting vector machine (SVM) and its variants such as SVR, seasonal SVM, and Online-SVM have been explored to improve traffic prediction performance due to their capabilities to generalize well and capture the high dynamics and sensitivity of traffic data [42,43,44]. Artificial neural networks (ANNs), with their advantages such as capability to work with multi-dimensional data, implementation flexibility, generalization ability, and good forecasting performance, have been applied to traffic prediction problems [45]. Kumar et al. applied an ANN to predict traffic volume using time information in addition to past traffic related data such as volume, speed, and density [46]. Kashi and Akbarzadeh used wavelet transformation to remove unimportant fluctuation from the flow signal and then an ANN to train on past data to predict future flow on different highways and locations [47].

Although these methods can model the non-linearity and extract spatial-temporal relationship in the traffic data to achieve better results than parametric approaches, they require significant prior domain knowledge and extensive preprocessing work such as feature engineering. With traffic density increasing, wide adoption of sensors and cameras, and popularization of GPS-enabled navigation apps for smartphones, the big data paradigm has emerged from transportation related data. Such data explosion introduces a problem well-known as the curse of dimensionality, which cannot be handled efficiently by traditional approaches [48,49]. To gain insight from big traffic data, deep neural networks have become popular in recent years to learn deep correlations inherent in data with little or no prior knowledge and need for manual feature engineering [13,50]. Stacked autoencoder models have been used to exploit temporal and spatiotemporal information on real-world or simulated datasets to predict traffic flow [5,51]. Recurrent neural networks (RNNs) especially LSTM have been used to predict traffic flow, speed, and congestion because of their built-in memory cells enabling learning temporal knowledge and thus being suitable for time-series analytics [52,53,54]. Convolutional neural networks (CNNs), with its special strength in extracting spatial correlations, have been adopted for traffic speed prediction on image generated from speed data [4,55]. Deep belief networks (DBNs) have also been used for traffic flow prediction due to their capability to learn effective representative features from data in an unsupervised way [50,56,57]. Furthermore, there have been research efforts which combine more than one kind of deep neural networks. Yu et al. [6] proposed a deep learning model named SRCN combining CNNs and LSTMs to capture and learn both spatial dependencies of different roads and long-term temporal dependency of each road and to predict traffic speed on a certain set of roads, using in-house synthesized road maps with floating car GPS data.

However, these attempts mainly focus on prediction of traffic related properties such as flow, speed, and time on a single road segment, several number of roads, or a small network region [21,58,59,60,61]. One major reason for lack of such studies of traffic congestion prediction is the challenge to obtain large data set. One recent work by Ma et al. [7] used the congestion information collected from the GPS data from taxi to model and predict traffic congestion evolution. However, this dataset has time intervals of 30 minutes and 60 minutes, which is too sparse for training models for real-world congestion prediction. It also spans only four weeks.

## 3. Methodology

In this section, we describe our accessible and general approach for obtaining and processing raw snapshots of traffic congestion maps from online map service providers. In our approach, traffic congestion data are extracted automatically using an image mask for highways and a customized map-reduce implementation is used to process and transform these extracted congestion data originally represented as pixels into float numbers. Our method can extract traffic congestion levels from raw traffic congestion maps or snapshots provided publicly and for free by online map and traffic service providers. Then we propose a deep neural network (DNN) architecture for traffic congestion prediction.

Inspired by research findings of computer vision and deep learning in motion prediction which estimate future trajectories of objects via sequences of scenes generated by itself [62], we first collect time series of snapshots of network-wide traffic congestion map from Washington State Department of Transportation (WSDOT) [17] with the help of web browsers and web crawlers, and then build a dataset from these snapshots. The approach can be easily extended to other online map service providers to build more datasets. This dataset is then used to train and back-test different deep learning models for congestion prediction. Based on the theory of city management grid modeling [63], we segment the transportation network covered by these snapshots into different grids. Each snapshot is divided into non-overlapping tiled 8 × 8 pixel grids where each grid corresponds to an area of about 80×80 m${}^{2}$ in the real world. Each grid has a congestion level calculated as the average of traffic congestion levels represented by all pixels in that grid.

### 3.1. Representation of Congestion Level of the Transportation Network

**Figure 1:**
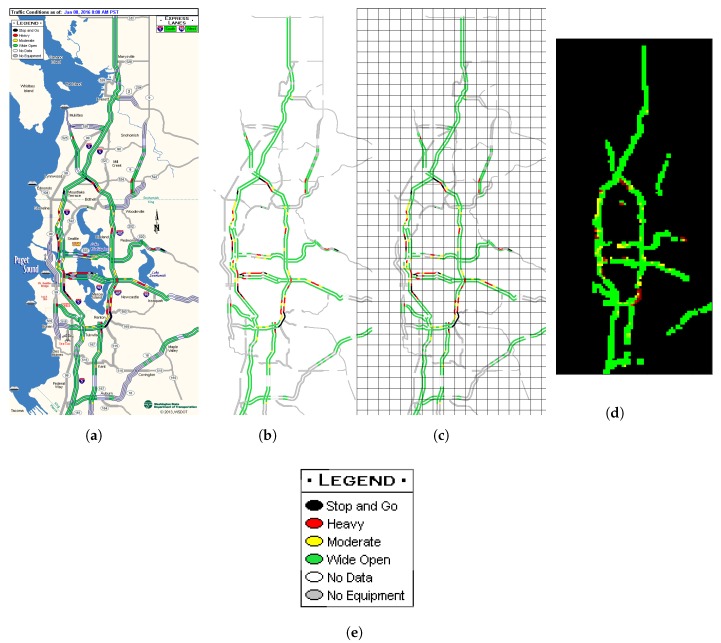
(**a**) A snapshot with traffic congestion levels from WSDOT. (**b**) Only the road network is retained after preprocessing. (**c**) The road network is segmented into grids. (**d**) The congestion of each grid is calculated, normalized, and colored for visualization. (**e**) Legend of congestion levels for Figure 1a as from WSDOT.

Since we want to forecast congestion levels inside every grid in a traffic network, we first retain only the road network and congestion conditions in the transportation network by removing all other pixels with the help of an image mask as shown by the transformation from Figure 1a to Figure 1b.

Then we segment network-only images into R×C grids, as is shown by Figure 1c. For each grid located at (i,j) where 1≤i≤R and 1≤j≤C, $\left\{c_{t,i,j}^{k}|1\leq k\leq 64\right\}$ denote the set of congestion levels as represented by all pixels in that grid at time *t*. Calculated from $c_{t,i,j}^{k}$ according to Equation (1), ${\bar{c}}_{i,j}^{t}$ denotes the averaged congestion level of the grid at the time *t*.

(1) $${\bar{c}}_{i,j}^{t}=\frac{\sum_{k=1}^{64}c_{t,i,j}^{k}}{\sum_{k=1}^{64}\left[c_{t,i,j}^{k}>0\right]}$$

Based on Equation (1), the final congestion level representation for the segmented road network at time *t* is expressed by the matrix in Equation (2), where *R* stands for number of grids latitudinally and *C* longitudinally. Figure 1d provides a colored visualization of a matrix representation as expressed by Equation (2). Each pixel in Figure 1d stands for an area of 80×80 m${}^{2}$ and is rendered according to a custom linear color map [64] generated from a sequence of RGB values ((0,0,0),(0,255,0),(255,255,0),(255,0,0),(139,0,0)).

(2) $$R_{t}=\left[\begin{array}{cccc} {\bar{c}}_{1,1}^{t} & {\bar{c}}_{1,2}^{t} & \cdots & {\bar{c}}_{1,C}^{t}\\ {\bar{c}}_{2,1}^{t} & {\bar{c}}_{2,2}^{t} & \cdots & {\bar{c}}_{2,C}^{t}\\ \vdots & \vdots & \cdots & \vdots \\ {\bar{c}}_{R,1}^{t} & {\bar{c}}_{R,2}^{t} & \cdots & {\bar{c}}_{R,C}^{t} \end{array}\right]$$

Suppose that we need to predict traffic congestion levels for the road network at time points in (t+1,t+2,⋯,t+h) where *h* is the prediction horizonand historical congestion levels in the time range (t-n,⋯,t-2,t-1,t) are used as inputs. When we arrange historical records of network traffic congestion representation by time, we get the time-series sequence $\left(R_{t-n},\cdots ,R_{t-2},R_{t-1},R_{t},R_{t+1},R_{t+2},\cdots ,R_{t+h}\right)$.

Because multiple time intervals are used as input for forecasting, the traffic congestion prediction task can be regarded as a time-series sequence prediction problem. When all grids in the network are predicted at the same time, it is known as the multi-dimensional sequence learning problem.

### 3.2. Temporal Features

Sequences of snapshots of traffic congestion levels across a road network delimited by a fixed time interval in chronological order are very similar to natural language sentences consisting of words separated by spaces. Future congestion levels might be affected by earlier congestion levels to varying degrees due to the temporal dependency inherent in traffic data. In our work we try to use such temporal correlation for prediction of future congestion levels.

### 3.3. Deep Congestion Prediction Network

**Figure 2:**
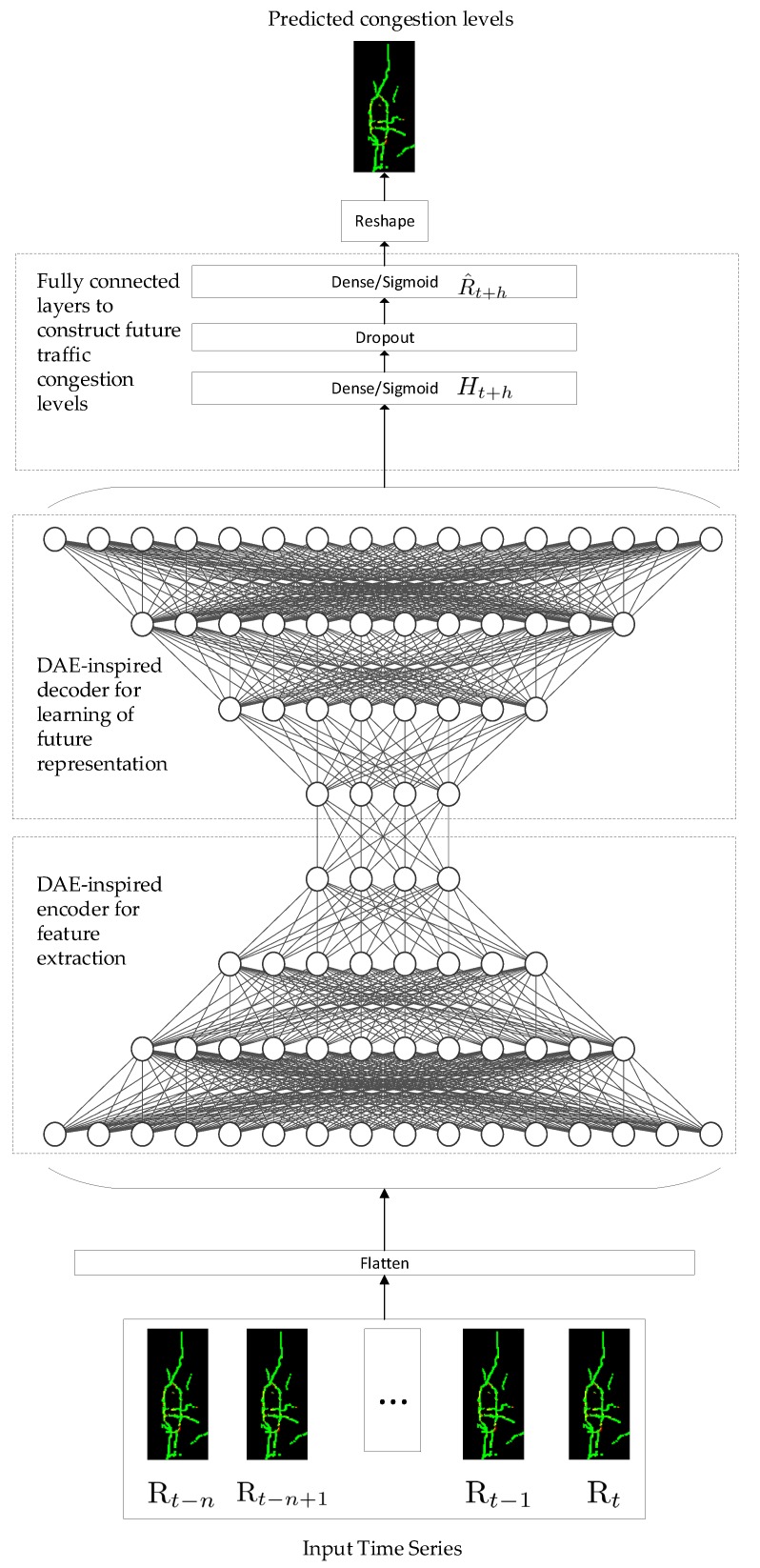
The architecture of our proposed deep neural network for traffic congestion prediction.

We propose to use a deep learning model titled Deep Congestion Prediction Network (DCPN) inspired by DAEs for transportation network congestion prediction. It is designed to learn and represent temporal features and correlations of traffic congestion levels among roads in the transportation network. Our proposed model consists of two components. The first component contains an encoder and a decoder. The encoder first obtains a vector representation of historical congestion levels of a transportation network and their correlations using four encoding layers. Next the decoder builds a representation of the congestion levels for a future time point using four decoding layers. The architecture of this first component uses symmetrical layers for the encoder and the decoder, while in DAEs the encoder and the decoder shares the inner most layer [22]. The second component of DCPN uses two dense layers to construct congestion levels for each grid in that transportation network at that future time point. These two dense layers take the output $F_{t}$ from the decoder in the first component and calculate a vector representation of predicted traffic congestion levels, as shown by Equation (3). $W_{h}$ and $b_{h}$ represent the weight matrix and the bias between the last layer of the DAE-like architecture and hidden dense layer, while $W_{o}$ and $b_{o}$ between the hidden dense layer and the output dense layer. To avoid overfitting, a dropout layer is added between the two dense layers. The prediction vector ${\hat{R}}_{t+h}$ has the same number of elements as each of input series and is further reshaped to have the same shape. Thus, in this way we train the proposed model from end to end. To integrate DAE-like architectures and dense layers for traffic congestion prediction, we propose to use the architecture in Figure 2 for DCPN to forecast traffic congestion on a network scale. A DCPN model includes one input layer, one flattened layer, one deep autoencoder, two dense layers with one dropout layer in between, and one reshaping layer.

(3) $${\hat{R}}_{t+h}=W_{o}\times \left(W_{h}\times F_{t}+b_{h}\right)+b_{o}$$

## 4. Experiments and Results Analysis

### 4.1. Data Source

Real-time congestion levels of roads in a transportation network are provided publicly by various online map service providers (such as Google map [18], Bing maps [20] and Autonavi map [19]) and transportation administration departments (for example WSDOT and Beijing Traffic Management Bureau [16]), and can be taken as or have been provided as a series of snapshots of different transportation networks including freeways, interchanges, intersections, ramps, and other elements. Figure 1a provides an example of a raw snapshot of a highway transportation network with traffic congestion levels in Seattle area from WSDOT. Each such raw snapshot is 557 ×1199 pixels in size, covers a large area of about 40 km × 96 km, and embodies congestion levels for mostly interstate and state highways. Service providers derive and infer congestion levels from different data sources, among which are mobility data generated collaboratively by mobile map apps running on smartphones used by commuters and sampling data from loop detectors.

We focus on congestion levels forecasting during morning rush hours from 07:00 a.m. to 10:00 a.m. on workdays. For that purpose, we collect snapshots of traffic congestion map for a transportation network in Seattle area in the aforementioned morning period from 1 January 2016 to 28 February 2017 and all missing data are left as is (a copy of these raw snapshots is available at https://github.com/senzhangcas/traffic-congestion with permission from the original data provider). After removing weekends and days without any snapshots, there are 301 days left. 283 of these days have 19 samples extracted from the time period between 7:00 a.m. and 10:00 a.m. with an interval of 10 min and 18 days have less samples due to missing data. Samples in the year 2016 are used as the initial train set and samples in the year 2017 are used as the test set via back-testing. We name the dataset built from criteria listed above as the Seattle Area Traffic Congestion Status (SATCS) dataset.

#### 4.1.1. Data Comprehension, Preprocessing and Representation

**Figure 3:**
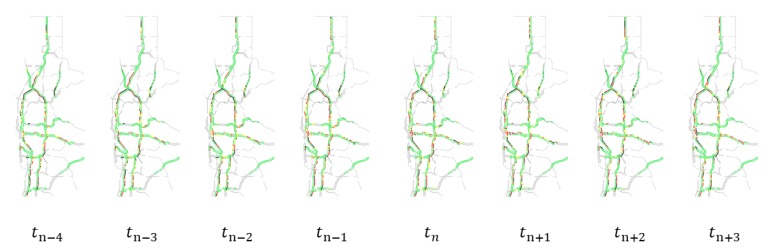
A sample of a sequence of images with only road networks organized chronologically for further transformations.

As can be seen from Figure 1a, traffic congestion status snapshots usually contain other elements such as signs and labels which are currently not considered for congestion prediction. We manually make a road network mask using image processing tools and develop shell scripts to filter out such unneeded elements and apply a preprocessing step to convert these raw snapshots to images with only the road network in concern (as shown in Figure 1b) and then these images are arranged chronologically into sequences (as shown in Figure 3) for further transformations as described in the next paragraph.

Each pixel in every image of such a sequence is converted to a float number based on its color (transparent, green, yellow, red, or black) for different congestion levels as shown by the legend in Figure 1e (non-road area, wide open, moderate, heavy, or stop-and-go). Inspired by the work in [65], to make the transformation easy and accurate, the HSL (Hue, Saturation, Lightness) color space is used. Furthermore, based on the theory of city management grid modeling, the 2D-matrix is first segmented (see Figure 1c) and then shrunk (see Figure 1d) via grids of 8×8 pixels according to Equation (1). After these preprocessing steps, sequences of 2D-matrices are provided as inputs to all the neural network models we experiment with.

### 4.2. Performance Comparison and Metrics

To evaluate performance of DCPN and its parameter configuration for congestion prediction, two state-of-the-art deep learning neural network-based models are selected as baselines for comparison. The first model is the SRCN neural network proposed to predict traffic speed using both spatial and temporal information [6]. This model consists of multiple CNN layers and LSTM layers. The second baseline is a ConvLSTM neural network consists of multiple ConvLSTM layers proposed by [26,66] for traffic prediction. ConvLSTM combines advantages of CNN and LSTM by using convolutional structures in both the input-to-state and state-to-state transitions. To perform a wider coverage of time horizons for comparison and analysis, we select three time intervals, namely 10, 30, and 60 min for prediction. We use two metrics—mean absolute error (MAE) and weighted mean squared error (wMSE) as defined in Equations (4) and (5)—to evaluate performance of traffic congestion forecasting in this paper. $c_{ij}^{t}$, ${\hat{c}}_{ij}^{t}$ and $w_{ij}^{t}$ denote the true traffic congestion level, the predicted level and the penalty weight at time *t* in the grid as designated by the point (i,j) in the 2D-matrix representation of the highway transportation network. To combat imbalanced distributions of different congestion levels, $w_{ij}^{t}$ is defined heuristically to put more penalty on incorrect prediction of non-smooth congestion levels. *W* and *H* denote numbers of grids along horizontal and vertical dimensions.

(4) $$MAE=\frac{1}{W\times H}\sum_{i=1}^{W}\sum_{j=1}^{H}\left|c_{ij}^{t}-{\hat{c}}_{ij}^{t}\right|$$

(5) $$wMSE=\frac{1}{W\times H}\sum_{i=1}^{W}\sum_{j=1}^{H}w_{ij}^{t}\times {\left(c_{ij}^{t}-{\hat{c}}_{ij}^{t}\right)}^{2}$$

### 4.3. Implementation of the DCPN Model

Previous work by Koesdwiady etc. has determined an optimized time lag of 120 min for traffic prediction [56]. Since samples in the dataset built by us have an interval of 10 min, we use 12 samples initially for DCPN’s parameter selection. We adopt a grid search of 5 configurations of number of hidden units for layers of DCPN to find a proper one. Results of the grid search are shown in Table 1. As can be seen, for prediction horizons of 60 min the 2nd configuration achieves minimum metric values for both wMSE and MAE which is followed very closely by the 1st one, while the 1st configuration perform the best in the 30 min case. With prediction horizon of 10 minutes, the 1st configuration archives minimum value on MAE and the 2nd one on wMSE. Further investigation using Welch’s t-test shows that there are no significant differences between both wMSE and MAE means produced by these two configurations, as shown in Table 2. However, the 1st configuration is more computationally efficient because fewer hidden units and connections are involved. So, we use the 1st configuration in our implementation of DCPN.

Furthermore, we experiment with both time lags of 120 and 110 min with the hope of reducing computing resource requirement. Results are shown in Table 3. Our experiment results show that the DCPN model performed better with the time lag of 110 minutes in terms of wMSE across all three prediction horizons. Thus, to reduce computational demand, for all three models and prediction horizons 11 historical samples before each testing point (as represented in 2D-matrices in Section 3.1) are used as inputs.

Detailed configuration of parameters for the DCPN model is listed in Table 4. The RMSprop optimizer [67] is used to train DCPN. The decay parameter is set to 0.9 and the learning rate is set to 0.001 and the batch size is set to a dynamic number equal to the count of samples in each day, which is provided by a custom data generator. We use a customized mean squared error algorithm with different weights for each congestion level as the loss function. Back-testing is deployed to predict traffic congestion for each workday during the morning rush hours in the year 2017 and samples from most recent 259 days before that day are used as the training dataset. Also, we use early stopping and a dropout layer to keep the model from overfitting [68].

For comparison, we also trained prediction models of two neural networks which can mine both spatial and temporal correlations according to existing literature: SRCN [6] and ConvLSTM [26]. We performed a grid search for both models. Results of the grid search show that the configuration [16, 32, 64, 128] achieves the best metrics for both models. So we adopt the SRCN model using the same configuration parameters as in [6] except the dropout rate is reduced from 0.2 to 0.1. For the ConvLSTM model, we use four stacked ConvLSTM layers with consecutive filter sizes set to 16, 32, 64, and 128; after each of these layers we add one max pooling layer, one activation layer with ReLU activation, and one batch normalization layer; finally one flatten layer, one dense layer, one dropout with a rate of 0.1, and one reshape layer are added to obtain the prediction result. For all three models, 11 historical samples before each testing point are used as inputs. In addition, all three models are implemented using the Keras deep learning library [69] on an Ubuntu 16.04 machine with 2 NVIDIA TITAN Xp Graphics Cards without a NVIDIA SLI connection.

### 4.4. Back-Testing

Instead of using cross validation for training and testing or fixed train/test split, we use back-testing for time-series prediction to collect metrics of applying models to the SATCS dataset for performance comparison. The widely used method of K-fold cross validation systematically splits the dataset into K groups and gives each group a chance to be a held-out test set. However, K-fold cross validation cannot be directly used with time-series data, because it assumes independence among the observations which is false for time-series data due to highly probable dependency among observations in temporal order imposed by the time dimension. Another common approach is to divide the samples into train and test datasets based on a specific time point as in [7], which does not meet the real-world requirement of daily or even near real-time prediction.

### 4.5. Results and Analysis

**Figure 4:**
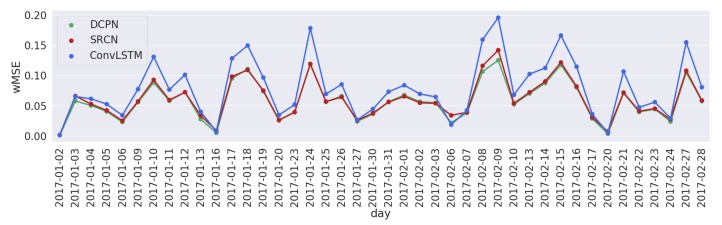
Daily total wMSE errors with a prediction horizon of 10 min on 42 days evaluated with back-testing.

**Figure 5:**
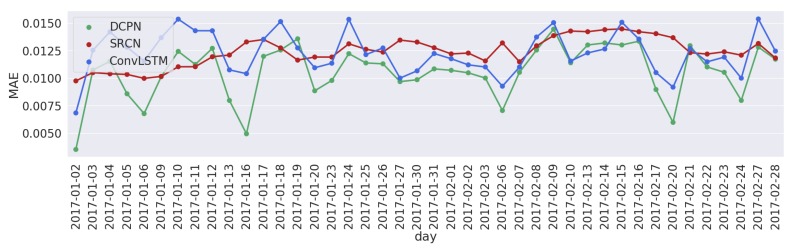
Daily total MAE errors with a prediction horizon of 10 min on 42 days evaluated with back-testing.

Figure 4 and Figure 5 show the daily total MAEs and wMSE errors for each of the 42 days from 2 January 2017 to 8 February 2017 of three compared models for network congestion prediction. A 10 min prediction horizon is used here and the daily total MAE and wMSE are calculated and shown in Figure 4 and Figure 5. As can be seen from these two figures, the DCPN model has the lowest prediction errors for most of the days.

For more details, Table 5 presents prediction performance per day for the whole transportation network by different models applied to the SATCS dataset across three prediction horizons. The DCPN model performs the best most of the time in terms of achieving the lowest values for both MAE and wMSE for the horizon of 10 minutes. Out of the 42 days for back-testing, it achieves the lowest values of MAE and wMSE for 32 and 33 days, respectively. While for horizons of 30 and 60 min, although SRCN is slightly better than DCPN when only MAE is considered, namely 22 against 20 days, and 21 against 21 days respectively for the two horizons, DCPN is overwhelmingly better than both SRCN and ConvLSTM by achieving the lowest values of wMSE on 41 and 40 days. The metric wMSE is pragmatically more significant and meaningful to travelers and administration departments because it concerns more about non-smooth congestion levels such as moderate, heavy, or stop-and-go ones through applying more penalties for incorrect prediction of such levels. So, it may be inferred that DCPN can be a stable candidate model for practical application of short-term traffic congestion prediction across all three prediction intervals. Furthermore, the averaged values of these two prediction error metrics—with the minimum averaged MAE of 0.0106, 0.0102 and 0.0095 and the minimum wMSE of 0.0579, 0.0536 and 0.0449 for horizons of 10, 30, and 60 min respectively—indicate that our proposed DCPN model achieves the best prediction performance. It can be seen that DCPN outperforms generally the other two on samples during the days for back-testing, suggesting that DCPN has a better generalization capability to new samples.

Both SRCN and ConvLSTM do not perform quite well for traffic congestion prediction on the SATCS dataset, while both have been reported to be able to use spatial information via CNNs [6,26]. On the one hand, characteristics of spatial structures of roads in transportation networks differ significantly from those of handwritten digits in various shapes by different people or images for the same category of objects. The spatial structure of roads stays unchanged while in the latter case each digit or object image has their own unique structure. On the other hand, roads in a metropolitan transportation network interact with each other differently than those in an urban transportation network do. In an urban transportation network, traffic congestion at a certain location can very likely cause congestion at other locations [6]. While in the SATCS dataset which covers the Seattle metropolitan area transportation network consisting of mostly interstate and state highways, we doubt that there exists such a spatial correlation of congestion among highways due to the vast area encompassed and sparse distribution of highways, so CNNs would have no spatial correlation to exploit to improve performance of congestion prediction. However it deserves further investigation to determine how and to what extent convolution operations in CNNs affect performance for congestion prediction in metropolitan transportation networks.

Also, the DCPN model is more computationally efficient than the other two in terms of computation time when applied to the Seattle area transportation network. Table 6 lists the training epochs and running time for the tested models. All three models are run on the same experiment equipment. Although DCPN needs more total number of epochs to train, it uses the least total amount of training time to converge, thus it is computationally most efficient and more suitable for real-world applications. We doubt that multiple convolutional operations inside both SRCN and ConvLSTM models applied to the Seattle metropolitan area transportation network consisting of mostly interstate and state highways would incur higher computing requirements. As an interesting phenomenon observed during our experiment, it suggests further work to investigate demand for computing resources by transportation networks with different sizes and distributions of roads.

**Figure 6:**
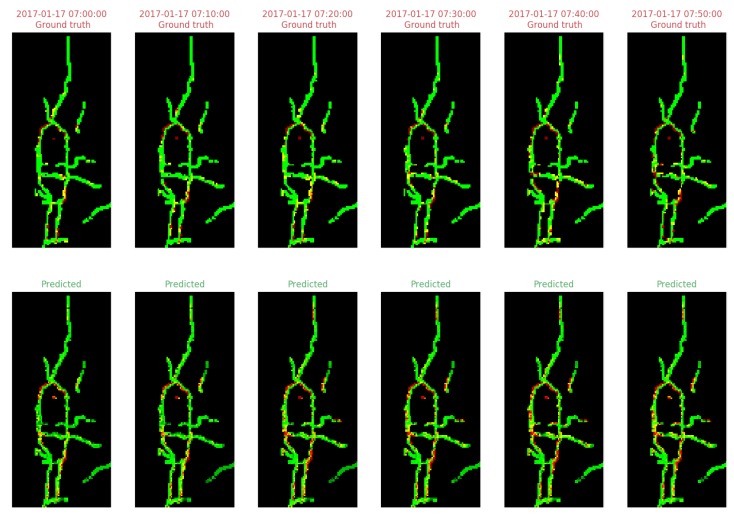
Ground truth congestion levels (first row) vs. predicted congestion levels (second row) on 17 January 2017 with a prediction horizon of 10 min.

As an example of the end-to-end prediction of traffic congestion levels by the DCPN model, Figure 6 shows both the ground truth and predicted congestion levels on the day 17 January 2017 with a prediction horizon of 10 min. Ground truth congestion levels at each time point are created using the same steps as described in Section 4.1.1. It can be seen that the DCPN model outputs visually intuitive prediction results in the same structure and form as the ground truth.

## 5. Discussion

Lack of large-scale traffic data related datasets with open access hinders research of intelligent transport systems. Data used in previous work—for example GPS and floating car data captured by taxis, images from surveillance CCTV cameras—either requires prior complex or special processes of application by users such as researchers and grant from authorities such as governmental agencies and companies. Nevertheless, there have been transportation network maps marked with congestion indicators or levels provided online for free by transportation administration departments and map service providers. For example, WSDOT has started providing such traffic congestion maps since at least as early as 2012 according to the Internet Archive [70]. To ease building of large-scale datasets for and to promote traffic congestion related research, we present a convenient and general workflow to create datasets for prediction of traffic congestion based on raw data from these free online service providers. Specifically, first we have made available an archived dataset of raw snapshots of the highway transportation network in the Seattle area from 1 January 2016 to 28 February 2017 as collected from WSDOT, most of which are no longer provided on the WSDOT website. Then based on this raw dataset, we build a dataset named SATCS and release it for future traffic congestion related research by us and others.

Even though now there is the SATCS dataset for traffic congestion research, some information is lost when congestion levels are represented in our current work. We must shrink matrix representations of congestion levels in the Seattle area highway transportation network using a grid-based scheme due to limitation imposed by GPU memory size of our current experiment equipment. As a result, each grid covers an area of 80 m × 80 m and the average of congestion levels represented by all pixels in a grid is used as the congestion level for that grid. During this shrinking process information is lost and geographical accuracy is reduced. It is yet unclear what information is lost and how loss of information affects prediction performance. We will deal with this information loss problem in our future work to predict traffic congestion at a finer granularity.

Furthermore, there is still room for improvement of computation efficiency. In the current representation of traffic congestion levels, most values equal 0.0 which is for grids containing no roads at all in the vast background area. However, DCPN still uses such values when training and testing, and thus incurs unnecessary computation. In our future work, we will try to exclude such background information to further improve efficiency.

## 6. Conclusions

In this work, we first propose an accessible and general approach to collect, transform and represent snapshots of road networks marked with congestion levels. We then apply it to build a dataset named SATCS for traffic congestion research. We develop a deep learning model DCPN by combining a DAE-inspired feature learning architecture and dense layers to learn representational features and temporal correlations from historical traffic congestion data for prediction of future congestion levels in a transportation network near the Seattle area, Washington state, USA. To evaluate the effectiveness of the proposed DCPN model for short-term traffic congestion forecasting, we compare its prediction performance with that of two state-of-the-art deep learning neural network models using the back-testing technique. Results over the SATCS benchmark dataset show that our proposed DCPN is more effective and computationally efficient for short-term traffic congestion forecasting.

This study focuses only on prediction of traffic congestion levels using traffic congestion snapshots from a single data source, and is limited by our experiment equipment. However, more extensive traffic forecast solutions are possible by covering travel time, volume, speed, and occupancy, and using other information such as weather conditions, which may be more accurate and meaningful for travelers, commuters, and administration departments. In future work, we will try to enhance computing capability of our experiment equipment to perform more thorough trials, experiment with snapshots from other service providers, and fuse multiple types of data from different sources, in order to build traffic forecast models for predicting aforementioned traffic condition related properties.

**Availability:** The Seattle area traffic congestion status dataset will be released publicly at https://mekhub.cn/Madsen/SATCS-dataset upon acceptance of this manuscript.

## Figures and Tables

**Table 1:** Comparison of prediction metrics by different configurations of DCPN. Minimum wMSE values marked in bold.

#	Prediction Horizon Averaged Metric Model Config	10 min wMSE	MAE	30 min wMSE	MAE	60 min wMSE	MAE
1st	512_384_256_128	0.058873	**0.010635**	**0.054298**	**0.010028**	0.045638	0.009572
2nd	640_512_384_256	**0.058357**	0.010737	0.054314	0.010125	**0.045414**	**0.009245**
3rd	768_640_512_384	0.061818	0.012796	0.058384	0.012838	0.049112	0.011893
4th	896_768_640_512	0.069279	0.016329	0.064761	0.016280	0.055138	0.016076
5th	1024_896_768_640	0.069227	0.016338	0.064663	0.016229	0.054909	0.015983

**Table 2:** Significance of difference between the top 2 configurations of DCPN using Welch’s t-test.

Prediction Horizon Averaged Metric Test Results	10 min wMSE	MAE	30 min wMSE	MAE	60 min wMSE	MAE
t stat	0.073076	−0.220181	−0.002431	−0.186772	0.038266	0.734073
p-value	0.941928	0.826291	0.998067	0.852313	0.969571	0.465075

**Table 3:** Comparison of prediction metrics using time lags of 120 and 110 minutes. Minimum wMSE values marked in bold.

Prediction Horizon Averaged Metric Time Lag (minutes)	10 min wMSE	MAE	30 min wMSE	MAE	60 min wMSE	MAE
110	**0.058730**	0.010705	**0.054224**	0.010130	**0.045305**	0.009293
120	0.058873	0.010635	0.054298	0.010028	0.045638	0.009572

**Table 4:** Configuration of parameters for DCPN.

Layer	Name	Channels	Shape
0	Inputs	1	(11, 149, 69)
1	Flattern	1	113,091
2	Dense (ReLU)	1	512
3	Dense (ReLU)	1	384
4	Dense (ReLU)	1	256
5	Dense (ReLU)	1	128
6	Dense (ReLU)	1	128
7	Dense (ReLU)	1	256
8	Dense (ReLU)	1	384
9	Dense (ReLU)	1	512
10	Dense (Sigmoid)	1	149×69
11	Dropout (0.1)	——	——
12	Dense (Sigmoid)	1	149×69
13	Reshape	1	(149, 69)

**Table 5:** MAE and wMSE by day of the whole network at different prediction horizons of 10, 30, and 60 min through back-testing. Best performance values for each day are marked with a bold typeface.

	10 min						30 min						60 min					
	**MAE**			**wMSE**			**MAE**			**wMSE**			**MAE**			**wMSE**		
**Day**	**SRCN**	**ConvLSTM**	**DCPN**	**SRCN**	**ConvLSTM**	**DCPN**	**SRCN**	**ConvLSTM**	**DCPN**	**SRCN**	**ConvLSTM**	**DCPN**	**SRCN**	**ConvLSTM**	**DCPN**	**SRCN**	**ConvLSTM**	**DCPN**
2017-01-02	0.0097	0.0068	**0.0035**	0.0017	0.0016	**0.0010**	0.0083	0.0069	**0.0034**	0.0014	0.0016	**0.0009**	0.0104	0.0072	**0.0067**	0.0037	**0.0023**	0.0025
2017-01-03	**0.0105**	0.0125	0.0107	0.0660	0.0651	**0.0576**	0.0115	0.0124	**0.0109**	0.0550	0.0590	**0.0543**	0.0103	0.0120	**0.0088**	0.0490	0.0500	**0.0483**
2017-01-04	**0.0104**	0.0142	0.0115	0.0527	0.0613	**0.0504**	**0.0103**	0.0123	0.0104	**0.0460**	0.0640	0.0471	0.0093	0.0137	**0.0088**	0.0387	0.0476	**0.0382**
2017-01-05	0.0103	0.0128	**0.0086**	0.0422	0.0525	**0.0404**	0.0112	0.0127	**0.0089**	0.0388	0.0491	**0.0381**	0.0110	0.0149	**0.0086**	0.0329	0.0405	**0.0315**
2017-01-06	0.0100	0.0116	**0.0068**	0.0251	0.0341	**0.0228**	0.0103	0.0111	**0.0071**	0.0274	0.0385	**0.0234**	0.0111	0.0131	**0.0077**	0.0264	0.0258	**0.0214**
2017-01-09	0.0102	0.0137	**0.0101**	0.0571	0.0773	**0.0557**	0.0110	0.0122	**0.0085**	0.0577	0.0787	**0.0531**	0.0120	0.0128	**0.0093**	0.0516	0.0538	**0.0447**
2017-01-10	**0.0110**	0.0154	0.0124	0.0927	0.1307	**0.0881**	0.0158	0.0141	**0.0118**	0.1051	0.1302	**0.0814**	0.0127	0.0146	**0.0115**	0.0917	0.1057	**0.0676**
2017-01-11	**0.0110**	0.0143	0.0112	0.0593	0.0765	**0.0582**	0.0167	0.0121	**0.0108**	0.0704	0.0795	**0.0508**	**0.0108**	0.0138	0.0111	0.0468	0.0534	**0.0395**
2017-01-12	**0.0119**	0.0143	0.0127	**0.0722**	0.1010	0.0725	0.0130	0.0146	**0.0124**	0.0900	0.1122	**0.0724**	**0.0115**	0.0131	0.0117	0.0812	0.0879	**0.0683**
2017-01-13	0.0121	0.0107	**0.0080**	0.0337	0.0399	**0.0277**	0.0100	0.0113	**0.0087**	0.0364	0.0375	**0.0259**	0.0103	0.0140	**0.0081**	0.0385	0.0391	**0.0285**
2017-01-16	0.0133	0.0104	**0.0049**	0.0089	0.0075	**0.0050**	0.0107	0.0093	**0.0058**	0.0089	0.0064	**0.0048**	0.0097	0.0122	**0.0044**	0.0067	0.0087	**0.0036**
2017-01-17	0.0135	0.0135	**0.0120**	0.0981	0.1282	**0.0951**	0.0124	0.0132	**0.0118**	0.1118	0.1262	**0.0912**	0.0110	0.0128	**0.0106**	0.0907	0.0983	**0.0752**
2017-01-18	0.0127	0.0151	**0.0126**	**0.1089**	0.1495	0.1106	0.0132	0.0162	**0.0122**	0.1318	0.1621	**0.1024**	0.0134	0.0142	**0.0107**	0.1169	0.1243	**0.0866**
2017-01-19	**0.0116**	0.0127	0.0136	0.0750	0.0965	**0.0738**	**0.0110**	0.0132	0.0112	0.0807	0.0943	**0.0665**	0.0112	0.0124	**0.0094**	0.0679	0.0755	**0.0533**
2017-01-20	0.0119	0.0109	**0.0088**	0.0263	0.0346	**0.0255**	**0.0079**	0.0124	0.0083	0.0292	0.0308	**0.0257**	**0.0072**	0.0108	0.0085	0.0208	0.0242	**0.0179**
2017-01-23	0.0119	0.0114	**0.0098**	**0.0391**	0.0517	0.0396	**0.0094**	0.0120	0.0098	0.0455	0.0519	**0.0373**	**0.0079**	0.0108	0.0088	0.0314	0.0337	**0.0264**
2017-01-24	0.0131	0.0153	**0.0122**	0.1191	0.1780	**0.1182**	**0.0131**	0.0146	0.0136	0.1330	0.1735	**0.1118**	**0.0114**	0.0141	0.0117	0.1261	0.1262	**0.0850**
2017-01-25	0.0126	0.0121	**0.0114**	0.0568	0.0693	**0.0563**	**0.0097**	0.0124	0.0123	0.0614	0.0691	**0.0546**	**0.0082**	0.0115	0.0092	0.0480	0.0512	**0.0397**
2017-01-26	0.0124	0.0127	**0.0113**	**0.0642**	0.0851	0.0658	**0.0100**	0.0122	0.0108	0.0697	0.0884	**0.0605**	0.0093	0.0118	**0.0090**	0.0578	0.0595	**0.0433**
2017-01-27	0.0135	0.0100	**0.0097**	0.0255	0.0264	**0.0240**	**0.0079**	0.0099	0.0082	0.0243	0.0300	**0.0219**	0.0079	0.0105	**0.0075**	0.0242	0.0253	**0.0187**
2017-01-30	0.0133	0.0107	**0.0099**	0.0377	0.0445	**0.0363**	**0.0085**	0.0105	0.0094	0.0312	0.0399	**0.0310**	0.0079	0.0103	**0.0073**	0.0262	0.0281	**0.0224**
2017-01-31	0.0128	0.0122	**0.0108**	**0.0560**	0.0731	0.0565	**0.0093**	0.0111	0.0107	0.0598	0.0693	**0.0493**	**0.0073**	0.0097	0.0085	0.0450	0.0527	**0.0403**
2017-02-01	0.0122	0.0118	**0.0107**	**0.0654**	0.0838	0.0675	**0.0105**	0.0116	**0.0105**	0.0723	0.0901	**0.0619**	**0.0086**	0.0109	0.0099	0.0618	0.0685	**0.0500**
2017-02-02	0.0123	0.0112	**0.0105**	**0.0547**	0.0694	0.0564	**0.0095**	0.0112	0.0105	0.0582	0.0662	**0.0518**	**0.0085**	0.0113	0.0093	0.0484	0.0502	**0.0408**
2017-02-03	0.0116	0.0110	**0.0100**	**0.0539**	0.0645	0.0548	**0.0095**	0.0107	0.0100	0.0550	0.0618	**0.0469**	0.0084	0.0103	**0.0079**	0.0401	0.0408	**0.0331**
2017-02-06	0.0132	0.0093	**0.0071**	0.0340	**0.0185**	0.0217	0.0123	**0.0095**	0.0097	0.0212	0.0189	**0.0108**	0.0127	0.0134	**0.0061**	0.0225	0.0308	**0.0208**
2017-02-07	0.0115	0.0110	**0.0105**	0.0390	0.0424	**0.0380**	0.0096	0.0102	**0.0079**	0.0361	0.0391	**0.0324**	0.0096	0.0106	**0.0074**	0.0299	0.0307	**0.0245**
2017-02-08	0.0129	0.0137	**0.0126**	0.1159	0.1591	**0.1064**	**0.0121**	0.0142	0.0125	0.1313	0.1812	**0.1046**	**0.0113**	0.0133	0.0122	0.1333	0.1674	**0.0943**
2017-02-09	**0.0139**	0.0150	0.0144	0.1417	0.1955	**0.1251**	0.0136	0.0157	**0.0133**	0.1562	0.2183	**0.1221**	0.0130	0.0139	**0.0123**	0.1464	0.1698	**0.1097**
2017-02-10	0.0143	0.0116	**0.0114**	0.0538	0.0679	**0.0522**	**0.0103**	0.0120	0.0105	0.0576	0.0718	**0.0488**	**0.0090**	0.0111	0.0101	0.0472	0.0546	**0.0411**
2017-02-13	0.0142	**0.0123**	0.0130	0.0722	0.1023	**0.0702**	**0.0110**	0.0124	0.0122	0.0809	0.0949	**0.0612**	**0.0095**	0.0118	0.0117	0.0694	0.0795	**0.0532**
2017-02-14	0.0144	**0.0126**	0.0132	0.0901	0.1123	**0.0873**	**0.0122**	0.0133	0.0134	0.1054	0.1381	**0.0891**	**0.0112**	0.0120	0.0115	0.1011	0.1137	**0.0815**
2017-02-15	0.0145	0.0151	**0.0130**	0.1214	0.1660	**0.1173**	0.0142	0.0152	**0.0124**	0.1366	0.1676	**0.1099**	0.0125	0.0141	**0.0120**	0.1213	0.1428	**0.0943**
2017-02-16	0.0142	0.0135	**0.0134**	0.0817	0.1144	**0.0802**	0.0118	0.0135	**0.0116**	0.0888	0.1150	**0.0733**	**0.0100**	0.0124	0.0112	0.0783	0.0895	**0.0643**
2017-02-17	0.0140	0.0105	**0.0090**	0.0306	0.0360	**0.0288**	**0.0074**	0.0101	0.0083	0.0308	0.0340	**0.0265**	**0.0072**	0.0100	0.0089	0.0289	0.0301	**0.0244**
2017-02-20	0.0137	0.0092	**0.0060**	0.0074	0.0052	**0.0034**	0.0101	0.0091	**0.0049**	0.0065	0.0070	**0.0041**	0.0112	0.0116	**0.0060**	0.0083	0.0096	**0.0048**
2017-02-21	**0.0123**	0.0127	0.0129	0.0716	0.1065	**0.0707**	**0.0101**	0.0125	0.0111	0.0769	0.1067	**0.0640**	**0.0090**	0.0109	0.0108	0.0630	0.0785	**0.0515**
2017-02-22	0.0122	0.0115	**0.0110**	0.0410	0.0472	**0.0396**	**0.0094**	0.0113	0.0096	0.0435	0.0519	**0.0341**	**0.0078**	0.0107	0.0089	0.0307	0.0363	**0.0274**
2017-02-23	0.0124	0.0119	**0.0105**	0.0451	0.0558	**0.0443**	0.0097	0.0114	**0.0094**	0.0435	0.0513	**0.0365**	**0.0075**	0.0102	0.0082	0.0306	0.0348	**0.0269**
2017-02-24	0.0121	0.0100	**0.0080**	0.0274	0.0291	**0.0236**	**0.0081**	0.0097	0.0085	0.0249	0.0308	**0.0218**	**0.0075**	0.0096	0.0113	**0.0195**	0.0235	0.0206
2017-02-27	0.0132	0.0154	**0.0128**	0.1078	0.1547	**0.1051**	0.0127	0.0166	**0.0120**	0.1142	0.1672	**0.0937**	**0.0110**	0.0134	0.0119	0.0910	0.1231	**0.0753**
2017-02-28	0.0118	0.0125	**0.0117**	0.0586	0.0804	**0.0576**	**0.0096**	0.0120	0.0113	0.0606	0.0800	**0.0522**	**0.0082**	0.0111	0.0118	0.0485	0.0604	**0.0427**
Average	0.0124	0.0123	**0.0106**	0.0603	0.0785	**0.0579**	0.0108	0.0121	**0.0102**	0.0647	0.0806	**0.0536**	0.0099	0.0120	**0.0095**	0.0558	0.0631	**0.0449**

**Table 6:** Computing resources for training with prediction horizons of 10, 30, and 60 min.

10 min			
	SRCN	ConvLSTM	DCPN
metric description			
total number of epochs to converge	876	719	823
total training time (s)	30,646.517	70,125.471	21,450.032
30 min			
	SRCN	ConvLSTM	DCPN
metric description			
total number of epochs to converge	757	631	845
total training time (s)	26,572.629	61,677.397	22,235.832
60 min			
	SRCN	ConvLSTM	DCPN
metric description			
total number of epochs to converge	769	690	795
total training time (s)	27,585.755	66,646.381	20,299.434
